# The building as a body: architectural perspectives in anatomy and the musculoskeletal system

**DOI:** 10.1016/j.ero.2026.02.004

**Published:** 2026-03-20

**Authors:** Yann Meudic, Andrea Hinojosa-Azaola, Dominique Le Nen, Alain Saraux

**Affiliations:** 1 Department of Rheumatology and pôle PHARES (Pharmacie, Recherche, Epidémiologie, Santé publique, Santé au travail), Université de Bretagne Occidentale (Univ Brest), CHU Brest, INSERM LBAI (U1227), LabEx IGO, Brest, France; 2 Department of Immunology and Rheumatology, Instituto Nacional de Ciencias Médicas y Nutrición Salvador Zubirán, Mexico City, Mexico; 3 Department of Orthopaedic Surgery, Université de Bretagne Occidentale (Univ Brest), CHU Brest, Brest, France; 4 Department of History of Science and Technology, Brest/Nantes, France

## Abstract

Since antiquity, the human body has inspired architecture both aesthetically and structurally. Vitruvius introduced proportion as a principle of harmony, whereas Leonardo da Vinci and Michelangelo studied anatomy to enrich artistic and architectural concepts. Their dissections and analyses of muscles, joints, and bones influenced ideas of stability, flexibility, and dynamic structures. Later, architects such as Gaudí, Eiffel, Le Corbusier, Parent, and Calatrava integrated biomimicry and biomechanical principles into their works, translating skeletal and muscular systems into innovative forms. Gaudí’s Casa Batlló and Sagrada Família evoke bones and tendons, whereas Eiffel’s Tower mirrors femoral trabeculae. Le Corbusier’s Modulor system formalised body-based proportions, and Calatrava explicitly referenced the spine and rib cage in his buildings. Beyond symbolism, modern ‘healing architecture’ demonstrates how spatial design impacts health and recovery. By bridging architecture and medicine, particularly rheumatology, biomimetic approaches highlight the musculoskeletal system as a lasting model for functional, inclusive, and therapeutic spaces.

## INTRODUCTION

Since the architectural art exists, the anatomy of the human body has been a model that has influenced, both aesthetically and structurally, the discoveries and improvements in built structures. Primitive humans used their own bodies as the primary system for measuring and proportioning their constructions [1]. During Antiquity, Marcus Vitruvius Pollio (c. 80–15 AD) drew inspiration from the anatomy of the human body to create innovative and functional spaces [2] and to introduce the notion of proportion, which in both art and the human body is synonymous with harmony and balance. He writes: “... just as in the human body there is a relationship between the elbow, the foot, the palm of the hand, the finger and the other parts, so in works that have reached their perfection, in members in particular make judge of the greatness of the whole work”. For example, the diameter of a column or the modulus of a triglyph makes one judge of the greatness of a temple” (Vitruvius).

Fascination with nature undoubtedly goes back as long as human existence itself. Biomorphic architecture, which draws from organic forms (animals, plants, and the human body), what is still called bio-inspiration, was prevalent in the Art Nouveau movement of the late 19th century [[3], [4], [5]]. The intention was to transcend the mimicking of natural forms and attempt to understand the principles that lie behind those forms and systems [6]. Among the many parts of the human body, rheumatology’s influence remains quite unexplored. However, the biomechanical principles governing the stability, flexibility, and resilience of the skeletal and joint systems find obvious parallels in architectural design. Although analogies between the human skeleton and load-bearing structures, or between joints and flexible architectural mechanisms, may seem intuitive, they have rarely been the subject of in-depth analysis.

Architecture, as a form of art, engages with fundamental questions of human existence in space and time, of the self and the world, interiority and exteriority, temporality and duration, life and death [1]. Rheumatology, on the other hand, offers a compelling lens through which to examine musculoskeletal structure, deformation, and reconstruction. It is an art in the sense that it is more artisanal than artistic, but can feed itself from the knowledge of the arts [7].

It is interesting to note that sources on this subject remain scarce. Beyond the fact that the topic was not sufficiently explored in the past, another possible reason is the traditional separation between architecture and medicine. Architects, focused on immediate aesthetic and technical concerns, did not systematically seek to incorporate biomechanical principles into their designs. Furthermore, at certain times, comfort and ergonomics were not prioritised, and the connections between the human body and building structures were rarely theorised. As a result, the lack of sources likely reflects a historical neglect of these intersections until recently, when concepts like biomimicry and tensegrity have made these links more visible and subject to study.

We present the connections between some of the most renowned architects, their work, and rheumatology, highlighting how the anatomical features of the musculoskeletal system have influenced architectural concepts from both aesthetic and technical perspectives. Additionally, we examine the importance of designing therapeutic and inclusive environments, and how spatial design can impact individuals living with chronic diseases.

Given that architecture is poorly represented in the medical literature, we conducted an exploratory, nonsystematic literature search in PubMed and Google, including all articles and books with no time frame published in English, French, or Spanish. Search terms combined ‘rheumatology’ or ‘musculoskeletal’ with ‘architecture’, ‘architecture and healing’, ‘tensegrity’, and ‘biomimicry’, with the aim of identifying relevant interdisciplinary literature rather than performing a comprehensive or systematic review.

### Anatomical insight as architectural inspiration

*Leonardo da Vinci (1452-1519)* stands as one of the most remarkable figures of the Renaissance, celebrated not only for his artistic works but also for his contributions to scientific research. Human anatomy held a central place among his many interests. Leonardo completed countless dissections of cadavers, allowing him to acquire a profound understanding of the musculoskeletal structures of the human body, compiled within a corpus of anatomical drawings almost entirely preserved at Windsor [8,9]. His lifelong fascination with anatomy profoundly influenced both his artistic and scientific explorations. Some articles have analysed and expanded upon this connection, highlighting the influence of his anatomical studies on diverse disciplines, including architecture [10]. His observations transcended art, extending to engineering and architecture, and offering innovative perspectives for building design, which may have been influenced by anatomical structures.

A particularly notable example of this interaction between anatomy and architecture is found in his studies of muscles, tendons, and bodily mechanics. By studying how muscles and tendons interact to enable movement, Leonardo created drawings that can be interpreted as research anticipating architectural concepts. These observations may have indirectly inspired a more fluid and functional approach to building design. For instance, his studies on the lever system and force transmission in the human body (Royal Library, Windsor Castle: RCIN 919026, verso; RCIN 919015, verso; RCIN 919101, verso) could translate into architectural structures that employ these mechanical principles to distribute loads and reinforce structural stability. These ideas are reminiscent of concepts found in modern structures, where architecture seeks to be organic and fluid, inspired by the human body to achieve more efficient and functional forms [11]. Another pertinent example in this context is Leonardo’s study of joints, particularly the knees and elbows. Da Vinci studied their functioning with remarkable precision (Royal Library, Windsor Castle: RCIN 919004, recto). By dissecting the movement of joints and their capacity to transmit forces, he developed mechanisms directly inspired by these movements in the design of mechanical devices, but also, by extension, in the design of architectural structures where load transmission and the balance of forces are essential (Royal Library, Windsor Castle: RCIN 919075). This can be seen in architectural structures that mimic, to a certain degree, these dynamic and articulated systems. The concept of joints providing stability while allowing a degree of mobility or flexibility could be transposed into building design, particularly in the use of articulated structures and modular supports.

These principles also influence modern architectural approaches, such as those of Santiago Calatrava, who employs biomimetic forms inspired by the human body to enhance the structural integrity of his works. Scholarly analyses have further explored these connections, reinforcing the idea that Leonardo’s anatomical insights played a role in shaping architectural thought. Leonardo da Vinci did not only study muscles and joints for their functionality; he also explored skeletal structures (Royal Library, Windsor Castle: RCIN 919012, recto). In his drawings of bones, particularly the spine and skull, he sought to understand the internal structure and how forces act on bones (Royal Library, Windsor Castle: RCIN 919007, verso; RCIN 919058, recto). This can be viewed as a metaphor for architectural construction, where materials and structures must bear loads while maintaining equilibrium. These studies may have informed his architectural vision, wherein the solidity and functionality of buildings are enhanced by a rigorous analysis of the forces acting on structures. This concept of a ‘solid’ yet ‘light’ structure, found, for example, in the design of arches and vaults, reflects the possible influence of biological principles on architecture. The parallels between Leonardo’s anatomical studies and architectural principles continue to be a subject of extensive academic discourse, demonstrating the enduring relevance of his interdisciplinary approach. Let us specify here that the metaphorical relationship between the human body and architecture is further explained by the fact that painters and sculptors, in Leonardo’s time, were also often architects. Although Leonardo da Vinci did not explicitly apply his anatomical studies to architectural works in the modern sense, his research on musculoskeletal structures had an indirect influence on architectural thought during the Renaissance and beyond.

His observations of how muscles, tendons, and bones work together informed a rethinking of building structures, integrating biomechanical principles to enhance stability and efficiency. This approach paved the way for more organic and functional architectural designs, inspired by nature and biomechanics, and continues to influence contemporary architects. Let us clarify that the metaphorical link between the human body and architecture is explained all the more by the fact that painters and sculptors, in the time of Leonardo, were often also architects. However, it is important to note that Leonardo’s contributions to architecture were more conceptual than practical. He did not directly create architectural works, although he did have plans to build a palace in Romorantin for King Francis I of France [12], but his ideas and studies served as a foundation for later architects and engineers to explore new possibilities in structural design. His influence can be seen in the work of later figures, such as Italian Renaissance architects and even modern architects like Santiago Calatrava (see below), who integrate biomimicry into their designs. Da Vinci’s profound understanding of anatomy and mechanics likely shaped the evolution of architectural thinking, even if he did not produce a significant number of built structures himself.

*Michelangelo Buonarroti (1475-1564)*, an emblematic figure of the Renaissance, profoundly influenced the history of art through his representation of the human body. Unlike many artists of his time who limited themselves to studying antique sculptures and live models, Michelangelo took his exploration further by participating in cadaver dissections. It is documented only by accounts that from his adolescence, he attended or practiced his first dissections at the convent of Santo Spirito in Florence, which allowed him to acquire an in-depth understanding of human musculoskeletal anatomy, unlike Leonardo, who studied all of anatomy like a scientist. He was known for his lifelong fascination with anatomy, which deeply influenced his artistic and architectural works [13].

An allusion to the analogy between anatomy and architecture is noted in Michelangelo’s hand in a letter dedicated to Cardinal Rodolfo Pio di Carpi: “Therefore, it is certain that the members of architecture depend on the members of man. Whoever has not been or is not a good master of figures, and above all of notomies, cannot understand them” [14]. “Michelangelo’s association with the human form was no longer a philosophical abstraction, a mathematical metaphor. By thinking of buildings as organisms, he changed the concept of architectural design from the static one produced by a system of predetermined proportions, to a dynamic one in which members would be integrated by the suggestion of muscular power” [15].

This intimate knowledge of the human body is clearly reflected in his architectural works. For example, the façade of the Church of San Lorenzo in Florence, designed by Michelangelo, exhibits an organisation and structure reminiscent of the musculature and proportions of the human body. Moreover, in the Sistine Chapel, researchers have suggested that certain shapes, such as the glowing mantle surrounding God and the angels in The Creation of Adam, could resemble the silhouette of the human brain, highlighting the influence of anatomy on his architectural conception [16]. This interpretation has been further explored in scholarly analyses, reinforcing the idea that Michelangelo integrated anatomical principles into his artistic vision, but this should be considered with extreme caution regarding attempts at overinterpretation, and remembering the advice provided by the art historian Daniel Arasse regarding the pitfalls of anachronism [17]. In short, Michelangelo transcended mere artistic representation by integrating anatomical principles into his architectural designs. His deep study of the human body not only enriched the aesthetics of his works but also introduced technical innovations that marked a turning point in Renaissance architecture.

### Biomimicry, tensegrity, and skeletal systems in modern architecture

Biomimicry is an emerging field of research that seeks to solve design problems by drawing inspiration from natural models, systems, and elements [18]. In architecture, biomimicry goes beyond using nature merely as aesthetic inspiration; it aims to apply natural principles to address functional challenges in the built environment. It encourages architects to envision buildings as living organisms designed for living beings [6,19].

Another concept drawn from nature is tensegrity, a structural principle based on the balance between components under tension and those under compression. Tensegrity applies not only to architectural designs such as domes, bridges, and towers, but also to the human body, particularly in the fascia, extracellular matrix, lungs, and vascular collagen. These biological systems form interconnected tensegrity networks that reflect the same mechanical principles [[20], [21], [22]].

The works of Antoni Gaudí and Gustave Eiffel exemplify how natural forms have inspired architectural innovation. Both pioneers integrated biomimetic and structural principles into their designs, long before these concepts were formally recognised.

*Antoni Gaudí i Cornet (1852-1926)*, a Catalan architect, was born into a family of artisans. From an early age, he demonstrated a remarkable ability to visualise 3-dimensional structures mentally. His introspective personality and profound appreciation for the natural world greatly influenced his architectural vision [23]. Some biographical accounts have suggested a possible association between Gaudí’s childhood illness and his later interest in nature, although this relationship remains speculative. At approximately 6 years of age, Gaudí reportedly suffered from an articular disease (possibly juvenile idiopathic arthritis or rheumatic fever), which limited his physical activity and resulted in prolonged periods at home. According to historical sources, during this time, he engaged extensively in the observation of natural forms, animals, and plants. Any influence of this experience on his later architectural approach, however, cannot be established and should be interpreted with caution [24,25].

As a master of organic architecture, he drew much of his inspiration from nature and human anatomy to design his buildings. His observation of bones, joints, and muscles allowed him to transpose biomechanical principles into innovative architectural structures. Through load-bearing and ornamental elements, he replicated the flexibility and strength of the human skeleton, creating works where architecture appears alive. In nature, bones achieve optimal structural balance by using minimal material, maximising resistance, and adopting hyperbolic shapes. When made from the same amount of material as traditional architectural columns, these forms are more durable than cylindrical ones [23]. This influence is particularly highlighted in architectural articles that establish a connection between his creations and the human body, notably in *Casa Batlló* and the *Sagrada Família*, where the transposition of anatomical forms reinforces the unique identity of his designs.

**Figure 1:**
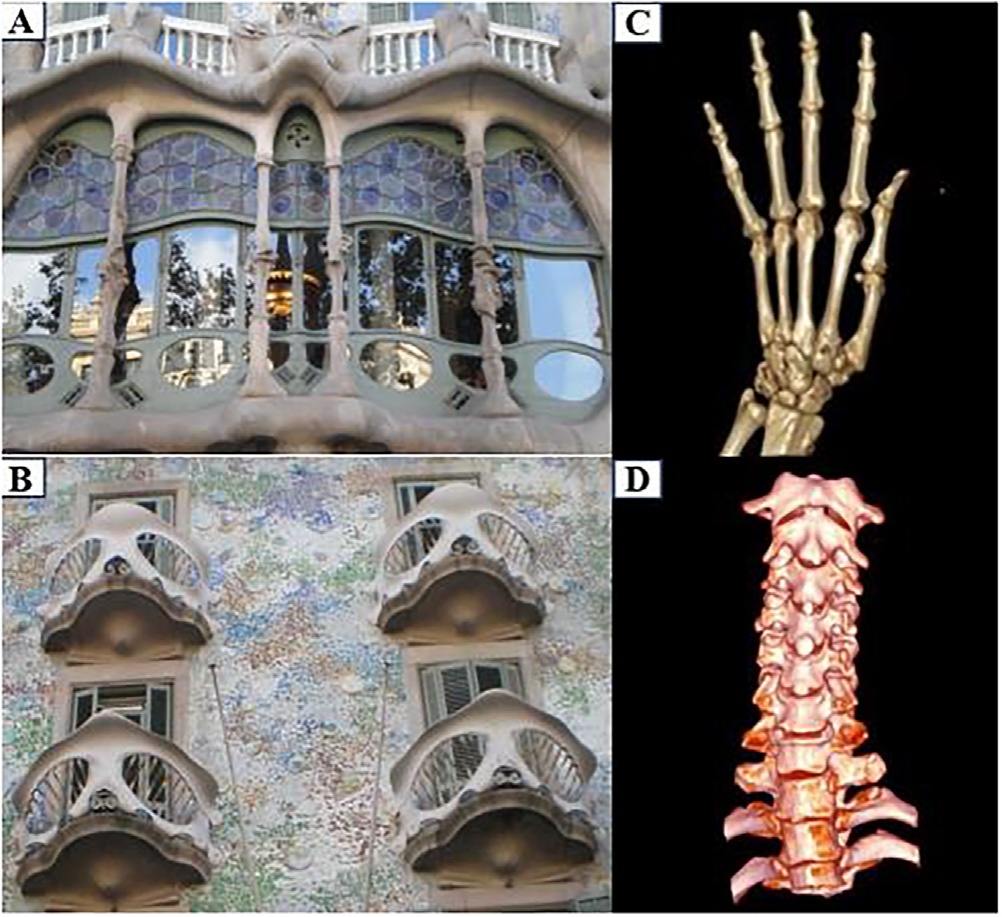
Analogy with the human skeleton in Gaudí’s *Casa Batlló*: The details on the façade (A and B) resemble phalanges (C) and vertebrae (D) © Photos Andrea Hinojosa-Azaola.

In *Casa Batlló* (1904-1906), the analogy with the human skeleton is evident in several architectural elements. According to morphological analyses of the building, the long columns on the facade resemble the shape of the femur and humerus, playing an essential load-bearing role. Similarly, the bases and capitals evoke vertebrae, suggesting a dynamic and segmented structure. The details continue with the marble balusters, which recall the fineness of phalanges, while the wrought iron railings imitate ribs, giving the facade an almost organic visual breath. These choices are not purely aesthetic: they reflect a structural consideration where each element contributes to the stability and balance of the building. Gaudí’s use of bone-like forms was one of the most striking elements of his Art Nouveau vision. These unusual structural features earned *Casa Batlló* the nickname ‘House of Bones’ [23] (Fig 1). Thus, *Casa Batlló* illustrates Gaudí’s ability to transpose biological principles into biomimetic and functional architecture, an idea widely discussed by various authors and architectural researchers.

**Figure 2:**
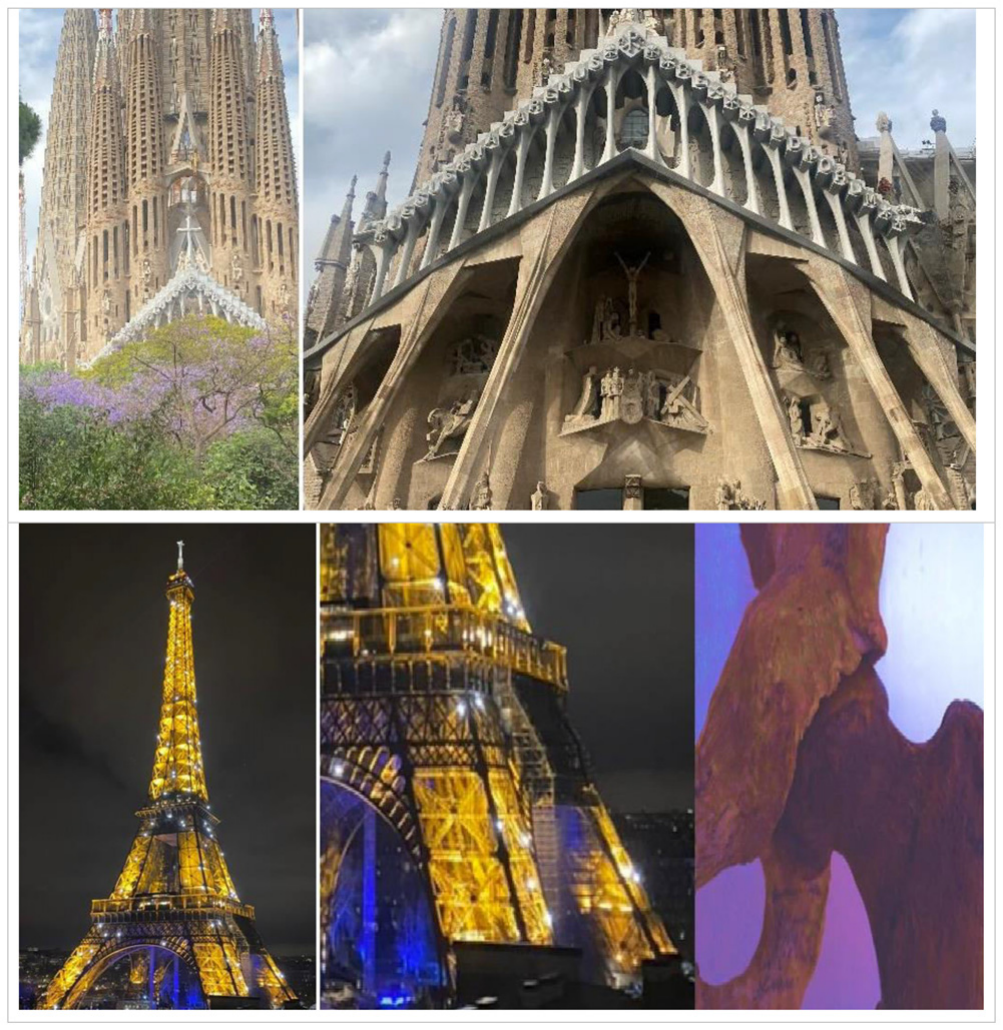
Examples of biomimicry. Upper: The inclined and twisted columns of the Passion Façade of the Sagrada Família (left), positioned in front of the sculptural group by Josep Maria Subirachs (right), evoke muscular and skeletal tension, intensifying the dramatic effect of the Crucifixion scene. Lower: The Eiffel Tower (left) and a comparison (right) between its base and a human femur—both featuring a lattice structure that provides strength and lightness. © Photos Alain Saraux.

The influence of the musculoskeletal system is even more pronounced in the *Sagrada Família*, particularly in the Passion facade and the central nave. The inclined and twisted columns of the Passion facade evoke muscular and skeletal tension, heightening the dramatic impact of the Crucifixion scene (Fig 2). Some observers, notably architectural historians, perceive tight tendons or exposed ribs, reinforcing the expression of suffering that Gaudí intended to convey.

Inside the basilica, the central nave is supported by branching columns that create a complex, tree-like network. This load-bearing structure resembles a forest but can also be interpreted as a human skeleton, an idea explored in the works of the Society of Architectural Historians. The system, inspired by trees, functions like a set of bones supporting the building, once again illustrating the integration of living principles into Gaudí’s architecture.

Comparisons between this structure and human anatomy are frequently discussed in specialised literature, emphasising the importance of this dimension in interpreting his work. Through *Casa Batlló* and the *Sagrada Família*, Gaudí demonstrated a deep understanding of human bone structure and its biomechanical principles. By incorporating references to the skeleton, joints, and muscles, he designed buildings that transcend conventional architecture and follow an organic, living logic. This biomimetic approach, far from being a simple stylistic device, reflects an advanced structural vision in which nature and human anatomy become the very foundation of architectural design.

*Gustave Eiffel (1832-1923)*, a French civil engineer, designed the Eiffel Tower, his most iconic work, for the 1889 World’s Fair in Paris. Now widely recognised as a global symbol of France, the tower is among the most visited monuments worldwide and is regarded as a landmark achievement in engineering. It has been suggested that aspects of the Eiffel Tower’s structural design may have been inspired by the internal architecture of the human femur, although this interpretation remains a matter of historical and scholarly debate [26] (Fig. 2).

The femur is lightweight yet capable of withstanding significant mechanical stress, an essential characteristic for architectural structures subjected to varying loads [9]. Internally, the femur contains a trabecular network of organised bone fibres that efficiently distribute forces, providing both strength and stability. Eiffel reportedly applied this biomechanical principle by incorporating a lattice framework into the tower’s structure, mimicking the crosshatched patterns observed in the femur’s internal architecture. Eiffel and his collaborators studied biological structures, particularly the way the femur’s fibres intersect in layers to resist compression, tension, and bending forces. By adapting this natural strategy, Eiffel minimised the tower’s weight while maximising its strength and wind resistance [27,28]. This analogy underscores how natural structures have inspired major architectural innovations, blending aesthetics with functional efficiency, a hallmark of biomimetic design.

### The body as structure: Visionary concepts and structural analogies

*Charles Édouard Jeanneret-Gris*, known as *Le Corbusier (1887-1965)*, was a Swiss-born, French-naturalised architect, designer, and painter, widely regarded as a pioneer of modern architecture. He was known for his definition of the home as ‘the machine for living in’ or ‘the machine for inhabiting’. With this, Le Corbusier emphasised not only the functional aspect of housing, but that this functionality must be directed towards living. He was fascinated by the then-emerging machines, especially automobiles and airplanes, viewing those with practical and functional designs as models for an architecture whose beauty would be based on practicality and functionality [29].

He sought to create a universal system of proportion that would harmonise architecture with the human body. Rooted in mathematics, anatomy, and proportion, this system reflects his belief that architectural dimensions should be derived from the human form rather than from abstract measurements. Developed between 1943 and 1955, the system, called the Modulor, was based on the proportions of an idealised human figure standing 1.83 m tall, with key reference points such as the height with an upraised arm (2.26 m) and the level of the solar plexus (1.13 m). Incorporating the Fibonacci sequence, the golden ratio, and human scale, the Modulor provides both practical and aesthetic guidance for design decisions, from furniture to entire buildings, by reconciling biology with architecture through the language of geometry [30]. The Modulor can be directly linked to the skeletal system, as it establishes a structural rhythm similar to the way bones provide a framework for the human body. Just as bones serve as a proportionate and organised structure to support movement, Le Corbusier’s proportional grid was intended to create spaces where the human body could function most efficiently. The concept mirrors how the skeletal system maintains balance and stability while also allowing fluid movement, principles that Le Corbusier applied to the design of buildings.

This idea is particularly evident in the *Unité d’Habitation*, where the dimensions of living spaces were dictated by the Modulor, ensuring ergonomic harmony between inhabitants and their built environment [30,31]. Just as bones maintain correct posture for muscles and joints, the Modulor defined an architectural ‘posture’ that optimised both comfort and functionality. By aligning architectural structures with human skeletal proportions, Le Corbusier reinforced the idea that the built environment should function as an extension of the body itself, a perspective further explored in numerous architectural studies [32].

Le Corbusier frequently compared the human body to a machine, emphasising its efficiency in weight distribution and equilibrium, principles that he sought to integrate into his architectural designs [33]. His fascination with biomechanics is evident in how he structured buildings to mimic the musculoskeletal system’s ability to handle stress and optimise load distribution. A particularly illustrative example is his *Palais des Soviets* project, in which he explicitly described architectural elements as analogous to bones, tendons, and muscles. He referred to ‘supporting bones’ and ‘muscular layers’, suggesting that a building’s structural framework should function like a skeleton: providing support while enabling flexibility and movement. This analogy influenced his practical approach to load-bearing structures. Just as the femur and tibia efficiently distribute the body weight through the lower limbs, Le Corbusier designed columns and beams to optimise force distribution within buildings. His *Dom-Ino* housing concept, with its free-standing skeletal structure, allowed for maximum flexibility in spatial organisation, similar to how the musculoskeletal system provides both support and mobility [30].

Moreover, his vision extended beyond individual buildings to urban planning. In *La Ville Radieuse*, he envisioned cities where structures were proportioned according to the Modulor, ensuring ergonomic flow and efficient weight distribution across large-scale environments [31]. This reflects how the human body channels forces through bones and joints to maintain balance and minimise strain. By treating architecture as a biomechanical system, Le Corbusier demonstrated how skeletal principles could be adapted to create spaces that are not only functional but also intrinsically aligned with the human body.

*Claude Parent (1923-2016)* was a visionary French architect and theorist known for radically rethinking architectural space through his concept of the ‘oblique function’, which challenged the conventional reliance on vertical and horizontal planes in modern architecture. His designs prioritised inclined surfaces, compelling the human body to engage dynamically with its environment, much like muscles and tendons working together to maintain posture and enable movement.

Architectural theorist Anthony Vidler drew connections between Parent’s work and human anatomy, further emphasising the biomechanical dimension of his designs [34]. The inspiration for the oblique function is deeply rooted in the biomechanics of the musculoskeletal system, as it challenges equilibrium, encourages movement, and disrupts passive occupation. When a person walks or stands on an inclined plane, their body naturally activates stabilising muscles, particularly in the legs and core, to maintain balance. This principle is evident in Parent’s architecture, where the floor itself becomes an active participant in human movement [35].

By replacing traditional flat surfaces with sloped ones, he created spaces that mimic how joints and muscles interact to adapt to different postures. For example, in his design for the Church of Sainte-Bernadette du Banlay, the inclined surfaces compel individuals to shift their weight continuously, echoing the biomechanics of walking or climbing. This architectural approach mirrors how the skeletal and muscular systems work together to absorb forces and distribute pressure efficiently. Just as tendons stabilise joints during movement, Parent’s sloped floors and walls encourage a constant redistribution of weight, fostering a more dynamic interaction between the body and architectural space [35].

*Santiago Calatrava (1951-present)*, a Spanish architect and engineer, has been deeply inspired by the human body due to his multidisciplinary training and profound interest in nature and biomechanics. Early in his career, he studied anatomy and human movement, perceiving in the body’s structure a harmonious relationship between aesthetics and functionality. One of his most explicitly body-inspired works is the *Turning Torso* in Malmö, Sweden. The design of this skyscraper was directly inspired by a sculpture created by Santiago Calatrava himself, an abstract, twisting human form. In the original sculpture, 7 cubes are set around a steel support to produce a spiralling structural effect. The building’s form is composed of 9 box units, shaped like cubes with triangular tips that rotate around a central core, resembling a spinal column. Calatrava has stated that he intended to express the spine’s ability to twist around its vertical axis. The interconnecting steel struts between the segments function similarly to ligaments, providing cohesion and flexibility to the structure [36].

Another anatomical source of inspiration for Calatrava is the rib cage. According to his official descriptions, he referenced the thoracic structure in the design of both the Oculus transportation hub at the World Trade Center in New York and the Quadracci Pavilion of the Milwaukee Art Museum. These designs evoke the rib cage both visually and structurally. The Milwaukee Art Museum’s movable brise-soleil, with its wing-like form, mirrors the symmetric and curved arrangement of ribs protecting the thoracic cavity, while also enabling controlled motion, much like the expansion and contraction of the chest during respiration. Similarly, the Oculus train station in New York features elongated, curbing steel beams that extend upwards and converge along a central spine, resembling ribs connected to a sternum.

These designs embody the balance of rigidity and flexibility, much like the human skeleton. Through his work, Calatrava demonstrates how the musculoskeletal system influences architecture, creating dynamic structures that interact with space and light.

### Ergonomic and therapeutic design: healing architecture

The relationship between architecture and medicine is reciprocal. In recent years, growing attention has been paid to how the built environment influences experiences of care and recovery in healthcare settings, a concept known as *healing architecture* [37]. Research has shown that facility design can play a pivotal role in promoting well-being and improving health outcomes. An example of this is a study that found patients recovering from cholecystectomy who were assigned to hospital rooms with a window view of a natural setting had shorter postoperative hospital stays, required fewer analgesics, and received fewer negative evaluative comments in nurses’ notes compared to patients in similar rooms with windows facing a brick wall [38].

Architectural design and elements of healing environments have been discussed in the rheumatology literature as potentially influencing patient well-being and quality of life, often through psychological and environmental pathways rather than direct therapeutic effects. As an example of this, HEMOVE Onlus, a nonprofit association, has promoted the creation of a multidisciplinary Task Group, which included rheumatologists, psychologists, and architects, with the aim of applying for the benefit of rheumatic patients the most modern technical skills available in the context of Environmental Psychology, including design and technology [39].

Therapeutic hospital environments incorporate features such as single-patient rooms, ambient conditions, natural sunlight, views of nature, and intuitive wayfinding systems [28]. Examples of this include the Maggie’s Centres designed by architects such as Frank Gehry, Zaha Hadid, and Thomas Heatherwick, among others. Located around the world, these dignified, human-centred spaces provide care and support for individuals with cancer and other chronic illnesses. The design of these healing spaces takes into account psychological, social, behavioural, and functional dimensions to actively support the healing process.

Table displays examples of drawings and buildings inspired by the musculoskeletal system, as well as examples of healing architecture created by renowned artists and architects.

**Table:** Examples of drawings and buildings inspired by the musculoskeletal system, as well as examples of healing architecture created by renowned artists and architects

Artist/architect	Drawings/buildings	Source
Anatomical inspiration		
Leonardo da Vinci (1452-1519)	Anatomical drawings of da Vinci’s dissections	https://arthive.com/fr/leonardodavinci/works/308457∼Human_skeleton
Michelangelo Buonarroti (1475-1564)	Anatomical drawings	https://wellcomecollection.org/works/cyg7xhf5
Biomimicry		
Antoni Gaudí (1852-1926)	*Casa Batlló*	https://rutadelmodernisme.com/ficha/casa-batllo-fr/
	*Sagrada Família*	https://commons.wikimedia.org/wiki/File:Sagrada_Familia_(voûte)_(5).JPG
Gustave Eiffel (1832-1923)	Eiffel Tower	https://www.batobus.com/fr/tour-eiffel
Structural analogies		
Le Corbusier (1887-1965)	The Modulor	https://blog.thal.art/fr/modulor-nombril-du-monde-de-le-corbusier/
	*Palais des Soviet* (project)	https://europeansectionlgm.typepad.fr/histoiregographiecollgeva/2013/11/larchitecture-totaliraire-à-venir.html
	*Ville Radieuse* (concept/project)	https://www.archdaily.com/411878/ad-classics-ville-radieuse-le-corbusier/51fadfbbe8e44ea2b0000010-ad-classics-ville-radieuse-le-corbusier-image?next_project=no
	Milwaukee art Museum	https://mam.org/info/architecture/quadracci-pavilion/
Claude Parent (1923-2016)	Sainte Bernadette du Banlay’s Church	https://www.nevers-tourisme.com/explorer/visiter-nevers/notre-riche-heritage/eglise-sainte-bernadette-du-banlay/
Santiago Calatrava (1951-present)	*Turning Torso*	https://en.wikipedia.org/wiki/Turning_Torso#/media/File:Turning_Torso2.jpg
	Oculus World Trade Center Transportation Hub	https://calatrava.com/projects/world-trade-center-transportation-hub-new-york.html
	Milwaukee Art Museum	https://mam.org/info/architecture/quadracci-pavilion/
Healing architecture		
Frank Gehry, Zaha Hadid, Thomas Heatherwick	Maggie’s Centres	https://architizer.com/blog/inspiration/collections/maggies-centres/

### Potential application and future directions

Rheumatic diseases may be understood as disruptions of the body’s architectural harmony, in which the balance between structure, flexibility, and load distribution is impaired. Inflammation, deformity, fibrosis, and ankylosis reflect a loss of structural adaptability, analogous to architectural systems that lose alignment, ultimately compromising coordinated musculoskeletal function.

Beyond its conceptual relevance, biomimetic principles derived from architecture have practical applications in rheumatology. Concepts such as load distribution and structural adaptation help interpret musculoskeletal damage and remodelling in inflammatory arthritis, spondyloarthritis, and osteoporosis. Therapeutically, this perspective supports function-oriented rehabilitation, controlled mechanical loading, and the design of healing environments that reduce stress and improve mobility. However, it is important to clarify the limits of analogy between anatomy and rheumatology.

Looking ahead, collaboration between science and architecture plays an important role in advancing biomimetic architecture. As clinical knowledge increasingly intersects with design, a new wave of architectural innovation will emerge, inspired by the structural and functional principles of the human body. This interdisciplinary dialogue promises to transform both fields, encouraging a deeper integration of biomechanics into architectural design. In the future, architecture inspired by anatomical insight may not only enhance structural performance but also respond to human needs, marking a significant evolution in the built world. Like the human body, a piece of architecture has to maintain its secret and mystery in order to ignite our imagination and emotions [1]. As American architect Louis Kahn once said: “leave a building be what it wants to be. It has its desires; if to the contrary, it defends itself, resists, you have to choose. From these successive choices, from the sketch stage up to the competition of works, results beauty and harmony” [40].

## CONCLUSION

To conclude, the human body, particularly its musculoskeletal system, has long served as a profound source of inspiration for architectural design. Aesthetic influences can be seen in the works of Antoni Gaudí, Le Corbusier, and Santiago Calatrava, while technical insights trace back to figures such as Leonardo da Vinci, Michelangelo, and Gustave Eiffel. These visionary creators have consistently drawn on the human body’s form and function, mimicking its structure, balance, and flexibility. In doing so, they have translated biomechanical principles into innovative buildings and spaces that are both visually striking and structurally efficient. Architecture and rheumatology share fundamental principles related to structure, movement, and adaptation, an intersection that continues to shape spatial design. As architects increasingly integrate biomechanical considerations into their work, this connection will likely deepen, ensuring the human body remains an essential reference point for the architecture of the future.

## CRediT authorship contribution statement

**Yann Meudic:** Writing – review & editing, Writing – original draft, Validation, Investigation, Data curation. **Andrea Hinojosa-Azaola:** Writing – review & editing, Writing – original draft, Visualization, Validation, Methodology, Investigation, Data curation. **Dominique Le Nen:** Writing – review & editing, Writing – original draft, Visualization, Validation, Investigation, Data curation. **Alain Saraux:** Writing – review & editing, Writing – original draft, Visualization, Validation, Supervision, Methodology, Investigation, Data curation, Conceptualization.

## Competing interests

All authors declare they have no competing interests.
